# The integrative knowledge base for miRNA-mRNA expression in colorectal cancer

**DOI:** 10.1038/s41598-019-54358-w

**Published:** 2019-12-02

**Authors:** Daša Jevšinek Skok, Nina Hauptman, Emanuela Boštjančič, Nina Zidar

**Affiliations:** 1 Institute of Pathology, Faculty of Medicine, University of Ljubljana, Ljubljana, Slovenia; 2 Agricultural Institute of Slovenia, Ljubljana, Slovenia

**Keywords:** Genetic databases, Genetics research

## Abstract

“miRNA colorectal cancer” (https://mirna-coadread.omics.si/) is a freely available web application for studying microRNA and mRNA expression and their correlation in colorectal cancer. To the best of our knowledge, “miRNA colorectal cancer” has the largest knowledge base of miRNA-target gene expressions and correlations in colorectal cancer, based on the largest available sample size from the same source of data. Data from high-throughput molecular profiling of 295 colon and rectum adenocarcinoma samples from The Cancer Genome Atlas was analyzed and integrated into our knowledge base. The objective of developing this web application was to help researchers to discover the behavior and role of miRNA-target gene interactions in colorectal cancer. For this purpose, results of differential expression and correlation analyses of miRNA and mRNA data collected in our knowledge base are available through web forms. To validate our knowledge base experimentally, we selected genes *FN1*, *TGFB2*, *RND3*, *ZEB1* and *ZEB2* and miRNAs *hsa-miR-200a/b/c-3p*, *hsa-miR-141-3p* and *hsa-miR-429*. Both approaches revealed a negative correlation between miRNA *hsa-miR-200b/c-3p* and its target gene *FN1* and between *hsa-miR-200a-3p* and its target *TGFB2*, thus supporting the usefulness of the developed knowledge base.

## Introduction

microRNAs (miRNAs) are approximately 21-nucleotide long RNA molecules involved in post-transcriptional regulation by binding to their mRNA targets^1^. RNA interference (RNAi) is one of the processes by which miRNAs regulate gene expression and is one of the most promising biological processes for the development of therapeutic by gene silencing. Each small RNA forms a sequence-specific, gene-silencing ribonucleoprotein, with specificity conferred by base-pairing between the small (guide) RNA and its target mRNA. There are many combinations of miRNAs and mRNAs, since miRNAs can bind completely to a complementary sequence of mRNAs, but can also bind incompletely (some nucleotides can have a mismatch complement base). The number of miRNA and mRNA pairs is increased because one miRNA can target multiple genes, and one gene can also be targeted by multiple miRNAs.

miRNAs are involved in the regulation of physiological processes, such as development, epithelial-mesenchymal transition (EMT), regulation of homeostasis and metabolism, etc. However, many miRNAs have been proven to be deregulated in many diseases, including cancer. They are involved in cell transformation from normal to malignant, including in colorectal cancer (CRC), which is one of the most common cancers and one of the leading causes of death worldwide^2^. There are many relationships among miRNAs and genes known to be involved in the initiation and progression of CRC^3^. Some of the known frequently inactivated genes in CRC are *APC*, *TGFBR2*, *TP53*, *SMAD4*, *PTEN*, constitutively activated *KRAS* or overexpressed *MYC* in CRC^3,4^. In addition to protein-coding genes and mRNAs, there are also miRNAs that are known to regulate CRC tumor-initiating cells, such as *hsa-miR-34a*^5,6^, *hsa-miR-106b*^7^, *hsa-miR-140*^8^, *hsa-miR-146a*^9^, *hsa-miR-183*^10^, *hsa-miR-200*^10^, *hsa-miR-203*^10^, *hsa-miR-215*^11^, *hsa-miR-302b*^12^, *hsa-miR-328*^13^, *hsa-miR-363*^14^, *hsa-miR-371*^15^ and *hsa-miR-451*^16^. In CRC, miRNA-mediated modulation thus regulates features of cellular transformation. However, many miRNAs also function downstream of these factors^3^. Furthermore, miRNA deregulation is also correlated to angiogenesis, proliferation and migration of cancer cells in CRC, thus contributing to cancerogenesis and invasion^17–20^. It is therefore crucial to increase our understanding of the role of miRNAs in CRC, and to discover novel therapeutic targets to improve diagnostic, prognostic and treatment options.

Bioinformatics and data-mining are being increasingly used to study a multi-omics approach to understanding the biology behind diseases. New technologies that enable large-scale data are being used to create databases. One of them is The Cancer Genome Atlas (TCGA), which is the largest cancer database with clinical and experimental genomics data. Another database is miRTarBase, which summarizes all low-scale and large-scale experimentally confirmed miRNA-mRNA pairs in various diseases^21^.

**Figure 1:**
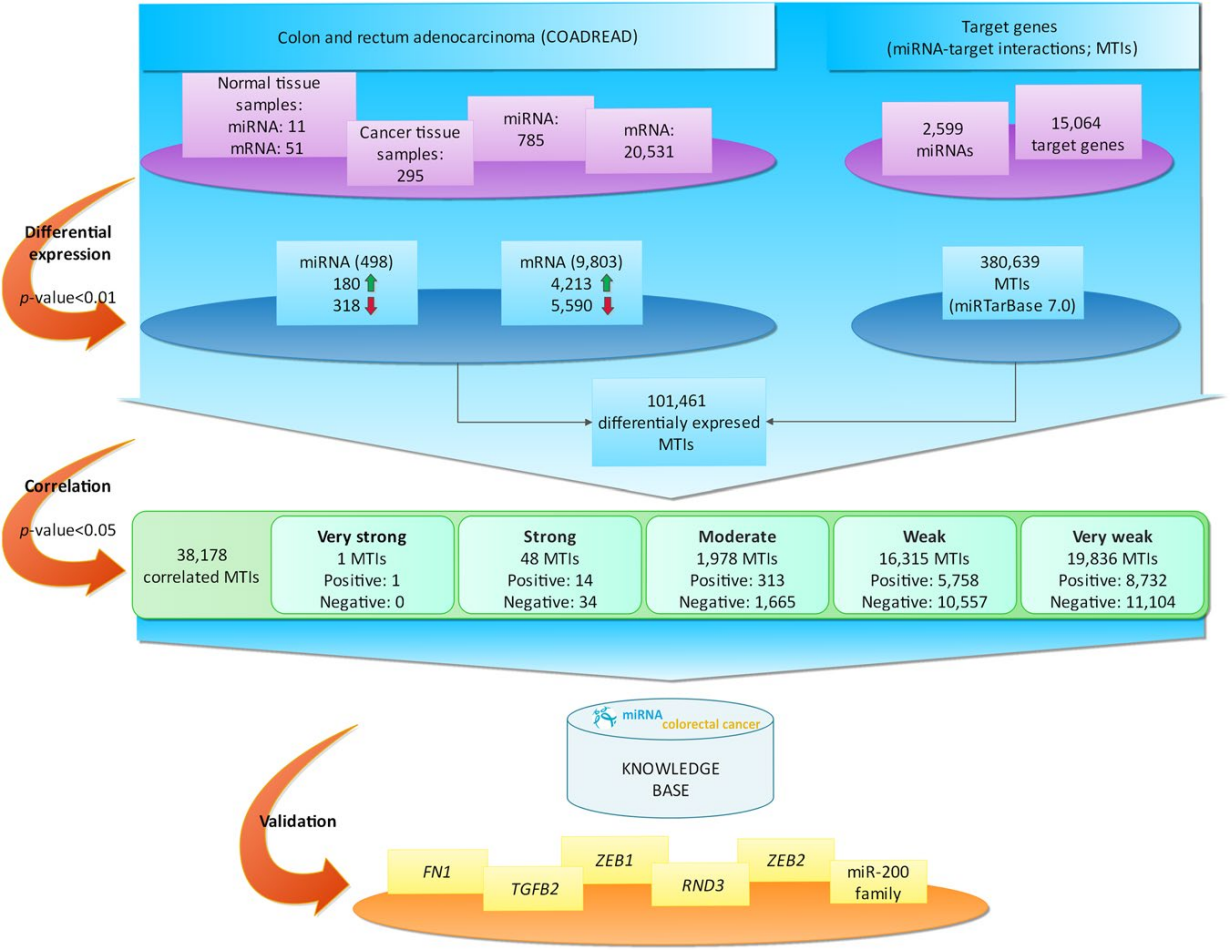
The workflow of the study. “miRNA colorectal cancer” was constructed from The Cancer Genome Atlas (TCGA) and from the miRTarBase databases. From TCGA, samples of Colon adenocarcinoma (COAD) and Rectum adenocarcinoma (READ) projects were used. Data from miRNA experiments obtained 11 normal samples, while data from mRNA obtained 51 normal samples. Number of cancer tissue samples with data from both miRNA and mRNA experiments was 295. Total number of miRNA in data from miRNA experiments was 785 and total number of genes from mRNA experiments was 20,531. Analysis of differential expression gave 498 differentially expressed miRNAs of which 180 were upregulated and 318 were down regulated. Differential expression analysis of mRNA gave 9,803 deregulated mRNA of which 4,213 were upregulated and 5,590 were down regulated. miRTarBase contains 2,599 miRNAs and 15,064 target genes from which there are 101,461 validated miRNA-target interactions (MTIs). Deregulated miRNAs and mRNAs were combined according to validated MTIs downloaded from miRTarBase and correlations were calculated between the pairs of miRNA and mRNA involved in one MTI. Correlation analysis revealed 38,178 correlated MTIs of which one MTI had very strong correlation, 48 MTIs had strong correlation, 1,978 MTIs had moderate correlation, 16,315 MTIs had weak correlation and 19,836 MTIs had very weak correlation. Results from differential expression and correlation analyses are combined in web application “miRNA colorectal cancer”. The knowledge base was further validated with wet-lab experiment, where we analyzed correlation between genes *FN1*, *SOX2*, *TGFB2* and *ZEB1* and *miR-200* family.

To identify mRNA-miRNA pairs that might serve as starting point to validate new potential prognostic and therapeutic targets, we performed an integrative analysis of miRNA-target gene interactions (MTIs) on high-throughput molecular profiling of 295 CRC samples from TCGA. We also experimentally validated 10 MTIs on our clinical samples. Figure 1 illustrates the workflow of the study. It consists of the following steps: data collection, statistics and data visualization, *in silico* screening for miRNA-mRNA interactions in CRC, and experimental validation.

The objective of this study was to help researchers in the field of miRNA-target gene interactions to decide which is the best miRNA or target gene to be validated in an experimental setting, whether or not they have previous experience in the field. For this purpose, we developed a freely available tool entitled “miRNA colorectal cancer”. The researchers can select a miRNA or gene they want to investigate further or they can use our tool even if they have no pre-selected miRNA or target gene.

## Results

Our knowledge base is based on high-throughput molecular profiling of colon and rectum adenocarcinoma samples from TCGA. We analyzed mRNA and miRNA expression in CRC samples and calculated the correlation coefficient for any of the known validated MTIs. The results of this meta-analysis are freely available through the web application “miRNA colorectal cancer”, which provides simple searching and reviewing of expression profiles of mRNA and miRNA and their correlation in CRC samples.

### Analysis of mRNA and miRNA expression in colorectal adenocarcinoma

To identify clinically relevant genes and miRNAs involved in CRC, the differential expression profiles of mRNA and miRNA were analyzed for CRC and normal samples. The analysis contained 20,531 protein coding genes, of which 9,803 were found to be differentially expressed genes (DEG) in CRC samples. A total of 4,213 DEGs were shown to be upregulated and 5,590 were shown to be downregulated in CRC. In the case of miRNA analysis, we found 498 differentially expressed miRNAs (DE-miRNAs) in CRC samples compared to normal tissue, 180 of miRNA were upregulated and 318 were downregulated in CRC.

### Target genes

The set of validated miRNA-target genes list obtained from miRTarBase contained 380,639 MTIs. These MTI pairs were obtained by combination of 2,599 miRNAs and 15,064 target genes. All of the validated 380,639 MTIs were combined with the set of analyzed DE-miRNA/DEG. The intersect between those sets of data revealed 101,461 MTIs in which both miRNA and mRNA were found to be significantly expressed in more than ten CRC samples (*p*-value < 0.01).

### Correlations between miRNA and their target genes

The correlation coefficient (R) between DE-miRNAs and their target genes was calculated using the Pearson correlation test. We analyzed 101,461 MTIs, with the condition that the miRNA-target gene had to be expressed in at least ten samples. This analysis provided the correlation coefficient for 38,178 MTIs that were significant, with a *p*-value of less than 0.05 (Supplementary Table 1).

### Using the “miRNA colorectal cancer” knowledge base

In this section, we will present examples of obtaining data from our “miRNA colorectal cancer” knowledge base through the web application. We will describe the usefulness of miRNA-mRNA expression and their interactions to understand their behavior of CRC better and to select appropriate candidate miRNAs or/and mRNAs for further experimental validation.

**Figure 2:**
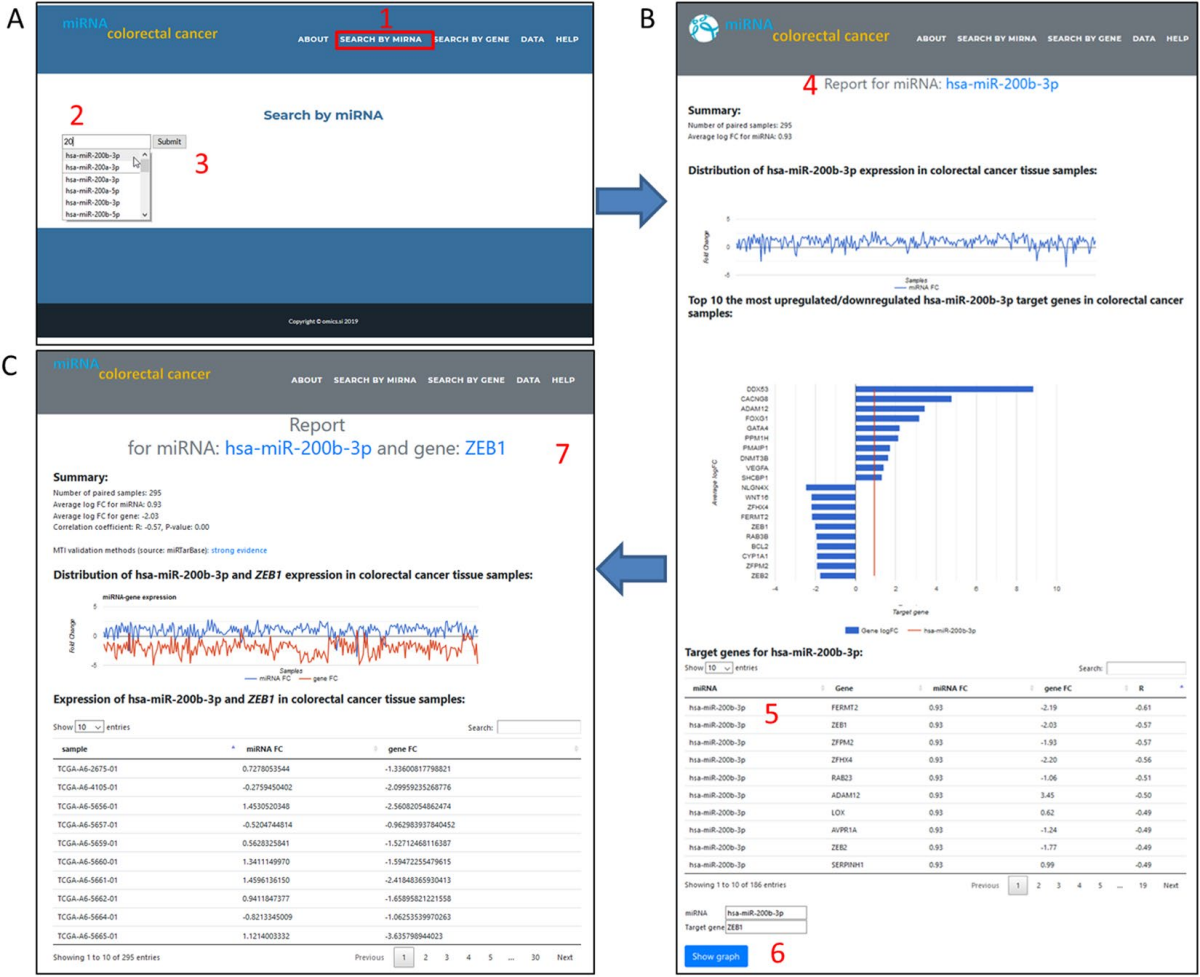
Example of “miRNA colorectal cancer” use. (**A**) Search by miRNA tab (1) allows users to start typing (2) the name of miRNA of interest or select it from a dropdown list (3). (**B**) Submit button opens a new browser tab with report for selected miRNA (4) in this case for *hsa-miR-200b-3p*. The report contains: summary of number of paired samples and average log fold change for selected miRNA (*hsa-miR-200b-3p*), distribution of selected miRNA (*hsa-miR-200b-3p*) in colorectal cancer tissue samples, top 10 most upregulated/downregulated miRNA (*hsa-miR-200b-3p*) target genes in colorectal cancer samples and list of target genes for selected miRNA (*hsa-miR-200b-3p*) (5). There are several options in table with target genes for selected miRNA (5): user can sort the table with arrows placed at the end of each column or can perform a search in the search form. By clicking miRNA-target gene in table (5) the queries for miRNA and target gene below are auto filled and user can get more information about this MTI by clicking on “Show graph” button (6), which opens another tab (**C**), the report for selected MTI – in this case *hsa-miR-200b-3p* and *ZEB1* gene) (7). MTI report contains: summary of number of paired samples, average log FC for miRNA, average log FC for gene, correlation coefficient with its *p*-value and MTI validation method (obtained from miRTarBase). The report also contains distribution of selected MTI (*hsa-miR-200b-3p* and *ZEB1*) expression in colorectal cancer tissue samples and expression of selected MTI (*hsa-miR-200b-3p* and *ZEB1*) in colorectal cancer tissue samples.

We can use two query interfaces to obtain data from the “miRNA colorectal cancer” web application: search by miRNA and search by gene. We present here search by miRNA option (1) (Fig. 2A). The user inserts an arbitrary miRNA (2) and presses the submit button (3). The result is opened in a new tab (4) and contains four parts (Fig. 2B). The first part of the report is a summary of the analyzed miRNA, which contains the number of paired samples and average logFC for this miRNA (it depends on user’s search input). The second part of the report is a distribution of the miRNA expression in the analyzed CRC tissue samples. The third part of this report shows the average logFC values for the ten (if there are ten or more target genes for this miRNA) most upregulated and ten most downregulated target genes for the searched miRNA. The fourth part of the report is a table view with all known miRNA-mRNA interactions for the searched miRNA. The table report enables a table search, as well as sorting individual columns by arrows at the end of each column. By clicking on the specific row, the form on the bottom of the page is auto-filled with the miRNA and gene names (5). A selection is submitted using the “Show graph” button (6). A new tab shows a new report for the specific MTI (previously selected) (Fig. 2C). The report contains: a) a summary of the analyzed miRNA and gene, which contains the number of paired samples, average logFC for the miRNA and gene, correlation coefficient and calculated *p*-value for selected MTI and MTI validation methods obtained from miRTarBase. The second part of the final report for the selected MTI is the expression in CRC tissue by samples shown as b) the distribution on a chart and c) a tabular view.

### Experimental validation

To validate our bioinformatics approach experimentally, we selected five miRNAs involved in EMT and their target genes based on literature and our previous and ongoing research in CRC^22,23^. We used our database to search for candidate MTIs which are among other functions involved in EMT (Table 1). For further experimental validation MTIs with *p*-value <0.01 were selected, including *FN1*/*hsa-miR-200b/c-3p*, *TGFB2*/*hsa-miR-200a-3p*, *TGFB2*/*hsa-miR-141-3p*, *RND3*/*hsa-miR-200b-3p*, *ZEB1*/*hsa-miR-200* family members and *ZEB2*/*hsa-miR-200* family members. For the selected MTIs, our bioinformatics and experimental results, including average logFC for miRNA and gene as well as correlation coefficient with its *p*-value are presented in Table 1 while the distributions of miRNA-target gene expressions are presented in Supplementary Figure 1.

**Table 1:** Candidate miRNA-target gene pair data from knowledge base and data from experimental validation.

MTI	Data from knowledge base				Experimental data		
	Average logFC miRNA	Average logFC gene	R	*p*-value	Average logFC miRNA	Average logFC gene	R
*FN1*/*hsa-miR-200b-3p*	0.93	−0.19	−0.49	<0.01	3.26	−1.01	−0.13
*FN1*/*hsa-miR-200c-3p*	−1.83	−0.19	−0.45	<0.01	0.90	−1.01	−0.45
*TGFB2*/*hsa-miR-200a-3p*	2.83	0.14	−0.32	<0.01	−1.17	−0.94	−0.19
*TGFB2*/*hsa-miR-141-3p*	4.16	0.14	−0.35	<0.01	0.78	−0.94	0.10
*ZEB2*/*hsa-miR-200a-3p*	2.83	−1.77	−0.48	<0.01	−1.17	0.91	0.15
*ZEB2*/*hsa-miR-200b-3p*	0.93	−1.77	−0.49	<0.01	3.26	0.91	0.31
*ZEB2*/*hsa-miR-200c-3p*	−1.83	−1.77	−0.45	<0.01	0.90	0.91	−0.06
*ZEB2*/*hsa-miR-141-3p*	4.16	−1.77	−0.54	<0.01	0.78	0.91	−0.04
*ZEB2*/*hsa-miR-429*	4.71	−1.77	−0.54	<0.01	1.73	0.91	−0.28
*RND3*/*hsa-miR200b-3p*	0.93	−0.45	−0.26	<0.01	3.26	−0.23	0.25
*ZEB1*/*hsa-miR-200a-3p*	2.83	−2.03	−0.57	<0.01	*ZEB1* was under limit of detection.		
*ZEB1*/*hsa-miR-200b-3p*	0.93	−2.03	−0.57	<0.01			
*ZEB1*/*hsa-miR-200c-3p*	−1.83	−2.03	−0.57	<0.01			
*ZEB1*/*hsa-miR-141-3p*	4.16	−2.03	−0.65	<0.01			
*ZEB1*/*hsa-miR-429*	4.71	−2.03	−0.62	<0.01			
*SOX2*/*hsa-miR-429*	4.71	−1.96	−0.11	0.08	Not experimentally validated MTIs.		
*CDKN1B*/*hsa-miR-200b-3p*	0.93	0.11	−0.03	0.56			
*CDKN1B*/*hsa-miR-200c-3p*	−1.83	0.11	0.10	0.09			
*CDKN1B*/*hsa-miR-429*	4.71	0.11	−0.09	0.14			
*ONECUT2*/*hsa-miR-429*	4.71	1.80	0.06	0.34			
*PTPN13*/*hsa-miR-200c-3p*	−1.83	0.49	−0.12	0.03			

logFC: logarithm of fold change, R: correlation coefficient.

As an example of MTIs involved in the regulation of cellular processes, there are several MTIs involved in EMT regulation. Genes *ZEB1* and *ZEB2*, the transcriptional factors of EMT, have a significant strong and/or moderate negative correlation with all five *hsa-miR-200* family miRNAs, which are the most studied EMT related miRNAs. In our *in silico* study, the correlation between *ZEB1* and *hsa-miR-200a/b/c-3p* was shown to be moderate (R = −0.57). Gene *ZEB1* (logFC = −1.33) is targeted by other two *hsa-miR-200* family members: *hsa-miR-141-3p* (logFC = 4.25) and *hsa-miR-429* (logFC = 5.03), with strong negative correlations. Another transcription factor of EMT, *ZEB2* (logFC = −1.77) has moderate negative correlations with *hsa-miR-200* family miRNAs, ranging from −0.45 to −0.54.

Gene *FN1* is a target gene for two *hsa-miR-200* family members (*hsa-miR-200b/c-3p*), with a moderate negative correlation (−0.49, −0.45, respectively), based on our bioinformatics study. Three other MTIs: *TGFB2*/*hsa-miR-200a-3p*, *TGFB2*/*hsa-miR-141-3p* and *RND3*/*hsa-miR-200b-3p* were selected with a statistically significant although weak negative correlation coefficient.

Our experimental data show the average logFC for gene *FN1* was −1.01 while the average logFC for *hsa-miR-200b-3p* and *hsa-miR-200c-3p* was 3.26 and 0.90, respectively. However, the correlation coefficient for MTI *FN1*/*hsa-miR-200b-3p* was very weak negative (R = −0.13) and for MTI *FN1*/*hsa-miR-200c-3p* we observed moderate negative correlation coefficient (R = −0.45). Gene *TGFB2* amplified in 15 clinical samples, with average logFC −0.94, while the average logFC for *hsa-miR-200a-3p* was −1.17 and for *hsa-miR-141-3p* it was 0.78. We observed a negative weak correlation for MTI *TGFB2*/*hsa-miR-200a-3p* (R = −0.19) and positive very weak correlation for MTI *TGFB2*/*hsa-miR-141-3p* (R = 0.10). Our experimental validation included also *ZEB1* and *ZEB2* of which *ZEB1* was under the limit of detection, while *ZEB2* was expressed with logFC of 0.91 and had a very weak positive correlation with *hsa-miR-200a-3p*, a weak positive correlation with *hsa-miR-200b-3p* and negative week/very weak correlations with *hsa-miR-200c-3p*, *hsa-miR-141-3p* and *hsa-miR-429*.

## Discussion

miRNAs are important post-transcriptional regulators of gene expression during development and physiology of human tissues. In general, miRNAs have an inhibitory effect on their targets. A prediction of active miRNAs is based on enrichment of differentially downregulated target genes of miRNAs^24,25^. Some bioinformatics tools have integrated miRNA-mRNA expression and correlation coefficients into their databases^21^ because the expression of RNA molecules is different in different tissues. Regarding CRC, we developed “miRNA colorectal cancer” web application, the largest knowledge base of the microRNA-target gene expressions and correlations in CRC, based on the largest available sample size from the same source of data. Therefore, the pre-calculated correlation coefficient between differentially expressed miRNAs and their targets is more reliable than that of other databases available.

Although our knowledge base is a standalone application for searching the information about miRNAs, mRNAs or MTIs, we attempted to test the application in experimental setting. There might be discrepancies between the application and experimental results, the most significant factor being the sample size, followed by the experimental method used and by the sample quality. We believe that with the same conditions for all samples, we could obtain the same results as in bioinformatics study if a large sample cohort was used. Moreover, when investigating miRNA and their target genes, the location of the sample used in experimental setting should be considered, for example different sections of tumor tissue could have different expression patterns.

For experimental validation, *miR-200* family and their target genes were used. miRNAs of the *miR-200* family are involved in several physiologic and pathologic processes, such as wound healing^26^, endometrial development during early pregnancy^27^, neurogenesis^28^ and inflammation^29^. The most documented process where *hsa-miR-200* family is involved is EMT, which is present also in CRC.

Many previous studies have revealed an association between gene *ZEB1*, a transcription factor of EMT, and CRC^10,30–35^. In humans, gene *ZEB1* is a target gene for 46 miRNAs^21^ and numerous studies indicate involvement of *ZEB1* in CRC. The complex formed by telomerase (hTERT) and ZEB1 binds directly to the E-cadherin promoter, inhibits E-cadherin expression and promotes EMT in CRC cells^32^. Gene *ZEB1* has numerous potential regulators beside miRNAs. MEF2D directly regulates transcription of *ZEB1* and facilitates histone acetylation at the *ZEB1* promoter in CRC cells^30,34^. Gene *ZEB1* not only promotes tumor cell dissemination but is also necessary for the tumor-initiating capacity of pancreatic and CRC cells^10^. In line with that, Quan *et al*. identified *ZEB1* as a target of *GRHL2* and suggested a reciprocal GRHL2-ZEB1 repressive relationship, providing a novel mechanism through which proliferation may be modulated in CRC cells^33^. Activation of *ZEB1* in CRC is also performed by overexpression of gene *SIX1*, which partly represses *hsa-miR-200* family expression^31^.

The *miR-200* family comprises *hsa-miR-200a*, *−200b*, *−200c*, *−141* and *−429*, and has been shown to play a role in cancer initiation and metastasis^22^. The majority of studies on the *miR-200* family are based on the assumption that they induce EMT in various diseases^36–40^. *miR-200* family members maintain epithelial cell integrity by suppressing EMT through direct inhibition of mesenchymal transcription factors such as ZEB1, ZEB2 and TGF-β^41^. Accordingly, we observed a correlation between upregulated miRNAs *hsa-miR-200a-3p*, *−200b-3p*, *−141-3p* and *-429* (Table 1, Supplementary Table 1) and their downregulated target gene *ZEB1*. Moreover, *hsa-miR-429* expression was upregulated in human CRC tissues, and high *hsa-miR-429* expression was significantly associated with tumor size, lymph node metastasis and poor prognosis^42^. Since the discovery of *miR-200* family functions in cancers, more detailed knowledge of this miRNA family may have therapeutic implications for the treatment of metastatic and drug-resistant tumors^37^.

However, the biological functions of intestinal miRNAs, not only the pathological aspect, are far more complex than initially suggested. The intestinal epithelium requires rapid changes in gene expression patterns to adapt to stress, and maintain epithelial homeostasis. miRNAs have emerged as fundamental regulators involved in many aspects of intestinal epithelial differentiation, architecture, and barrier function, as well as cellular self-renewal and inflammation, with specific miRNAs that promote intestinal homeostasis. Impact of miRNAs on the gut microbiota also plays an important role in intestinal homeostasis^43,44^.

miRNAs are also involved in regulating immune response and influence the expression of immune-related genes in intestine. They play important role in modulating the balance of resolution of inflammation, preventing tissue damage^43^. The most studied miRNAs whose targets are related to immune pathways are *hsa-miR-155*, *hsa-miR-146a*, cluster *hsa-miRs-17∼92*, and *hsa-miR-181a*. Many of these miRNAs target genes in Jak/Stat NF-κB and Akt pathways, which are immune response pathways^45,46^.

**Figure 3:**
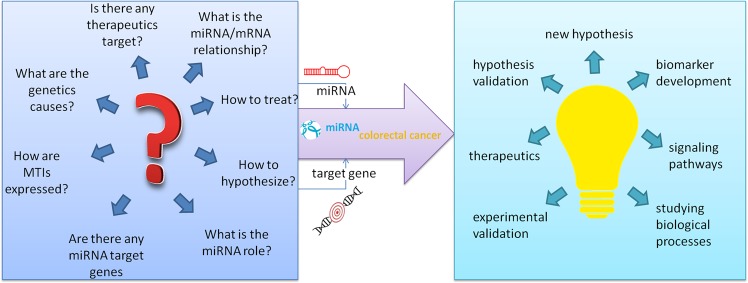
“miRNA colorectal cancer” as an integral part of bioinformatics process. How “miRNA colorectal cancer” can serve as a starting point in testing more targeted hypotheses and designing experiments using optimal miRNA, gene or both loci for further researches and analyses.

We believe that our knowledge base can assists researchers by providing step-by-step guidance on the formulation of a new research questions in the field of miRNA and mRNA expression profiles, their interactions and role in CRC (Fig. 3). Moreover, “miRNA colorectal cancer” knowledge base can also serve researchers as a starting point in testing more targeted hypotheses and designing experiments using optimal miRNA, gene or both loci for further researches and analyses.

## Conclusion

We performed *in silico* screening for miRNA-target gene interactions in CRC and presented them through an integrative knowledge base. We experimentally validated five genes and five miRNAs. These results can be applied not only for the experimental validation of MTIs involved in CRC, but also to contribute to our understanding of the biological processes and cellular mechanisms of organogenesis and cell differentiation. Our finding of a negative correlation between miRNA *hsa-miR-200b/c-3p* and *hsa-miR-200a-3p* and their target genes *FN1* and *TGFB2*, respectively, by both bioinformatics and experimental approaches, strongly supports the usefulness of the developed knowledge base. Collectively, our approach is efficient for choosing the appropriate candidate miRNA target genes in CRC and may provide an opportunity to discover and validate novel genes and cellular mechanisms as potential prognosis and/or diagnosis markers and therapeutic targets in CRC cancerogenesis.

## Methods

The “miRNA colorectal cancer” web application contains results of differential expression and correlation analyses of miRNA and mRNA data available from TCGA. The results are available through web forms within the newly developed tool.

### Data pre-processing

Data were obtained from the colon adenocarcinoma (COAD) and rectum adenocarcinoma (READ) project of TCGA from the Broad GDAC Firehose portal (https://gdac.broadinstitute.org/). We selected available RNAseqV2 and miRNAseq data, both performed on an Illumina HiSeq platform. A total of 295 samples that had both RNAseqV2 and miRNAseq data were selected. Additionally, 11 normal samples for the miRNAseq experiment and 51 normal samples for the RNAseqV2 experiment were selected. In both mRNA and miRNA experiments, level 3 data were used, which contain normalized gene counts and read per million mapped miRNA isoforms, respectively (Supplementary Table 2). For miRNAseq, we used data of calculated expression for each individual miRNA sequence isoform observed. For mRNAseq, we used RNASeqV2, which uses a combination of MapSplice and “scaled estimate” (RSEM) to determine expression levels. RNAseqV2 data contains a normalized read count, which represents the upper quartile normalized RSEM count estimates.

In tumor samples, values of normalized read counts were transformed to logarithmic values. In normal samples, normalized read counts were averaged through the samples and the averages were then transformed to logarithmic values. The logarithmic values of the average of normal samples were subtracted from each tumor sample logarithmic value, to obtain the logarithmic value of fold change for each tumor sample.

In miRNA level 3 data, we removed the stem-loop, precursor and unannotated reads, and summarized all reads belonging to a single isoform. The counts for each isoform in each sample were transformed to logarithmic values. For normal tissue samples, we also removed stem-loop, precursor and unannotated reads, summarized the reads for each isoform and averaged the isoforms before making the transformation to logarithmic values. The logarithmic values of the average of normal samples were then subtracted from logarithmic values of each tumor sample to obtain logarithmic values of fold change.

### Probes and genes

Target genes for miRNA were obtained from miRTarBase Release 7.0 (http://mirtarbase.mbc.nctu.edu.tw/php/index.php) (Sept. 13, 2019)^21^, and overlapped with mRNAseq data. The set of miRNA-target gene pairs contained 2,599 miRNAs and 15,064 targets. Further analysis was applied to 380,639 MTIs, downloaded from the miRTarBase database. The coordinates of protein-coding genes were obtained from Ensembl, release 98^47^, while the mature miRNA coordinates were obtained from miRBase Release 21^48^. The nomenclature of genes was unified according to the HUGO Gene Nomenclature Committee (HGNC; http://www.genenames.org/)^49^.

### Statistical analysis

The analysis was performed in R/Bioconductor software packages (http://www.bioconductor.org). A linear model fit was used for determining the significantly differentially expressed miRNA and mRNA in cancer compared to normal tissue, constructed in the “limma” package separately for mRNA and miRNA^50^. The threshold for the adjusted *p*-value was set to 0.01.

Pearson correlations were calculated using the mRNA and miRNA logarithm of fold change (logFC). The function was constructed using the “sigr” package in R language^51^. The Pearson correlation test was used to estimate the correlations between miRNAs and their target genes, with the *p*-value set to 0.05.

### Knowledge base development

The data from our study are stored in a relational database management system, MySQL (http://www.mysql.com). The data from the database is retrieved through the web interface and developed using HTML, CSS, JavaScript and Apache web server. Interactive charts were created by Google chart tools (https://developers.google.com/chart/) using a combination of programming languages PHP Hypertext Preprocessor (PHP) and MySQL.

### Experimental validation

#### Clinical samples

Our validation study was comprised of 30 patients with CRC. Tumor samples were collected during surgical colectomy of patients diagnosed with primary CRC, as well as samples from macroscopically normal colon mucosa from the same patient (at least 20 cm away from the tumor). Tissue samples were formalin-fixed, paraffin-embedded (FFPE) and stained with hematoxylin and eosin (HE). All the samples were analyzed as paired tumor samples, CRC sample and corresponding normal mucosa.

#### RNA isolation

RNA was isolated with the AllPrep DNA/RNA FFPE Kit (Qiagen), according to the manufacturer’s recommendations. RNA quantity and quality were determined spectrophotometrically by NanoDrop ND-1000 (Thermo Fisher Scientific).

#### Reverse transcription and qPCR experiment

Gene expression levels were determined using TaqMan based qPCR (Thermo Fisher Scientific), which was performed on the RotorGene system (Qiagen). Total RNA was reverse transcribed, 60 ng using the One Taq RT PCR Kit (NEB) according to the manufacturer’s instructions for mRNA quantification and 10 ng using the TaqMan microRNA reverse transcription kit (Thermo Fisher Scientific) according to the manufacturer’s instructions for miRNA quantification. For mRNAs quantification, the resulting cDNA was pre-amplified using a TaqMan PreAmp Master Mix Kit (Thermo Fisher Scientific). Relative levels of miRNAs and mRNAs were determined from diluted cDNA and pre-amplified cDNA, respectively, using qPCR and appropriate TaqMan probes. The expression levels were calculated after efficiency correction (based on the results of serial dilution) relative to four endogenous controls *hsa-miR-1247b* and *RNU6B* for miRNAs and *IPO8* and *B2M* for mRNAs.

### Ethics approval and consent to participate

The responsible ethics committee (Slovenian National Medical Ethics Committee) approved the reported investigation, which was carried out in accordance with the principles of the Helsinki Declaration.

## Supplementary information

Supplementary material info

Supplementary Table 1

Supplementary Table 2

## Data Availability

The knowledge base is freely available through a web interface at https://mirna-coadread.omics.si.
