# Supplementary material for: The integrative knowledge base for miRNA-mRNA expression in colorectal cancer
Source: Sci Rep. 2019 Dec 2;9:18065. doi: 10.1038/s41598-019-54358-w (PMC6889159; doi:10.1038/s41598-019-54358-w)
Supplement: Supplementary file 1 — Supplementary material info [file 41598_2019_54358_MOESM1_ESM.pdf]

Electronic Supplementary Information

For

The integrative knowledge base for miRNA-mRNA expression in colorectal cancer

Daša Jevšinek Skok[1,2§*], Nina Hauptman[1§], Emanuela Boštjančič[1] and Nina Zidar[1]

[1]*Institute of Pathology, Faculty of Medicine, University of Ljubljana, Ljubljana, Slovenia*

[2]*Agricultural Institute of Slovenia, Ljubljana, Slovenia*

[§] These authors contributed equally to this work

[*] Corresponding author: dasa.jevsinekskok@kis.si

A

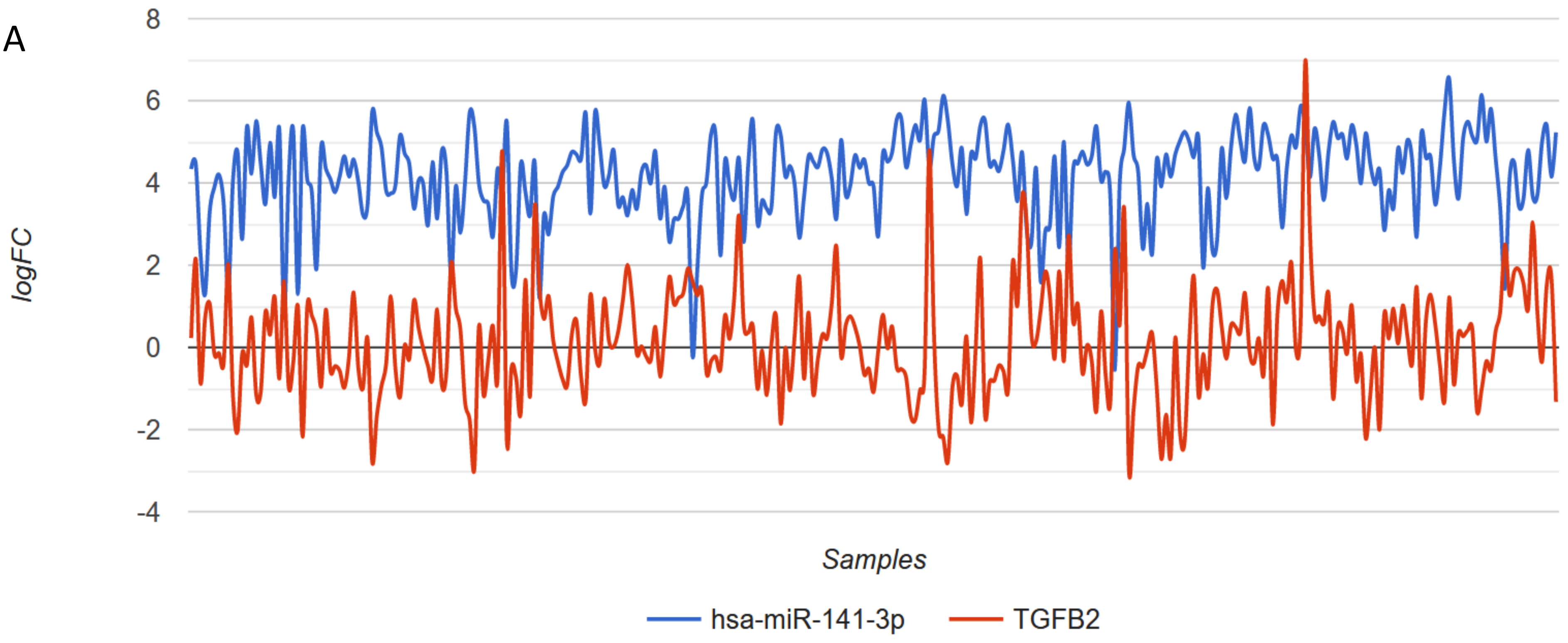
8
6
4
2
0
-2
-4
logFC
Samples
hsa-miR-141-3p
TGFB2

B

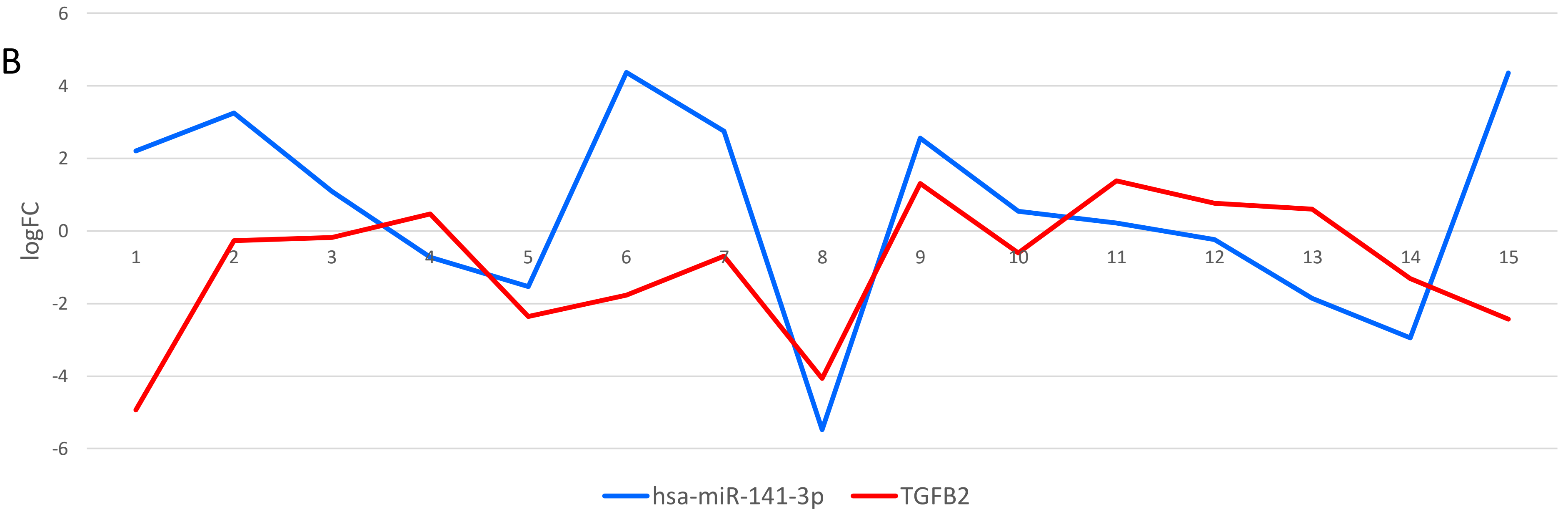
6
4
2
0
-2
-4
-6
logFC
1
2
3
4
5
6
7
8
9
10
11
12
13
14
15
hsa-miR-141-3p
TGFB2

A

B

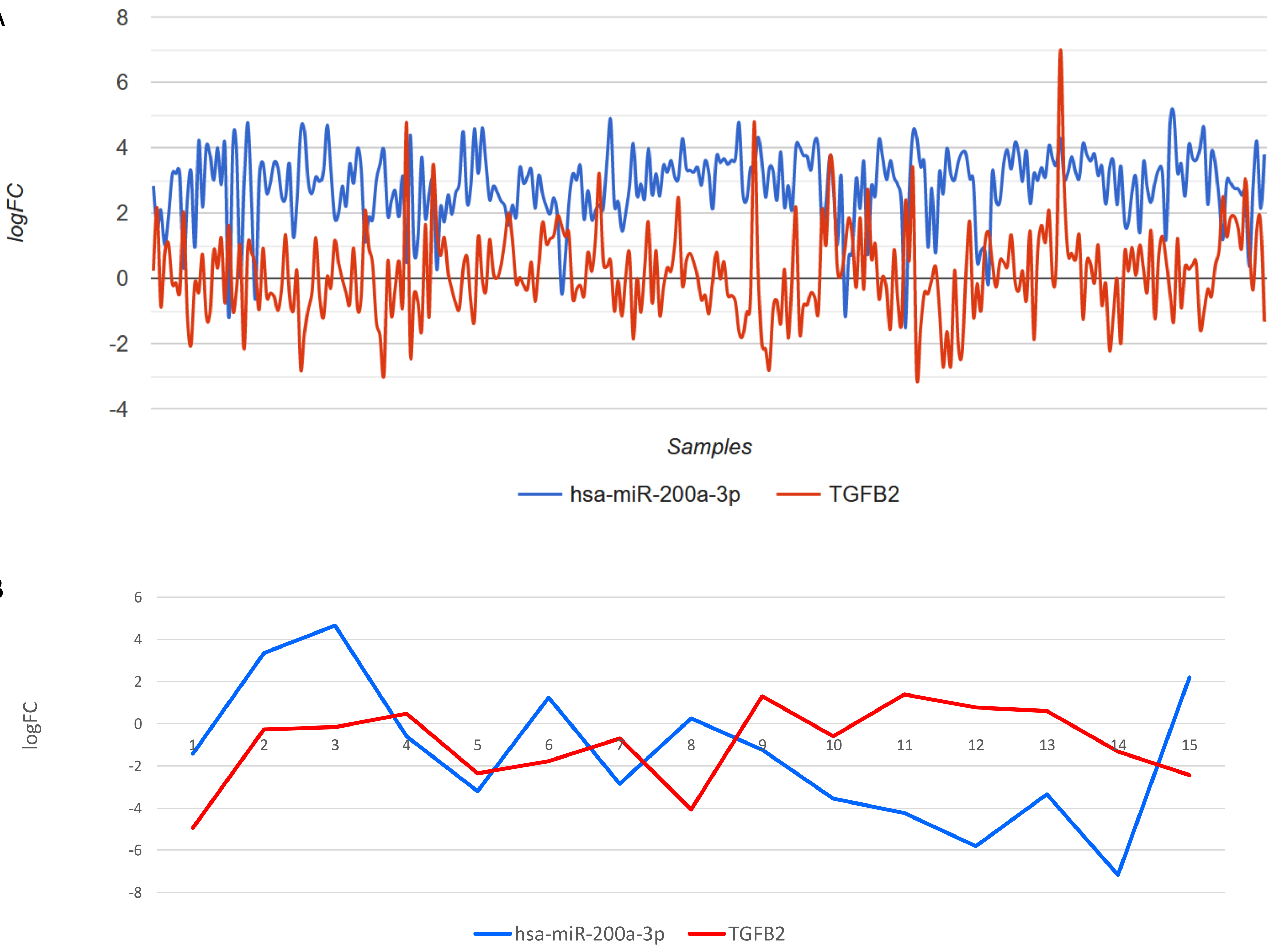
8
6
4
2
0
-2
-4
logFC
Samples
hsa-miR-200a-3p
TGFB2
6
4
2
0
-2
-4
-6
-8
logFC
1
2
3
4
5
6
7
8
9
10
11
12
13
14
15
hsa-miR-200a-3p
TGFB2

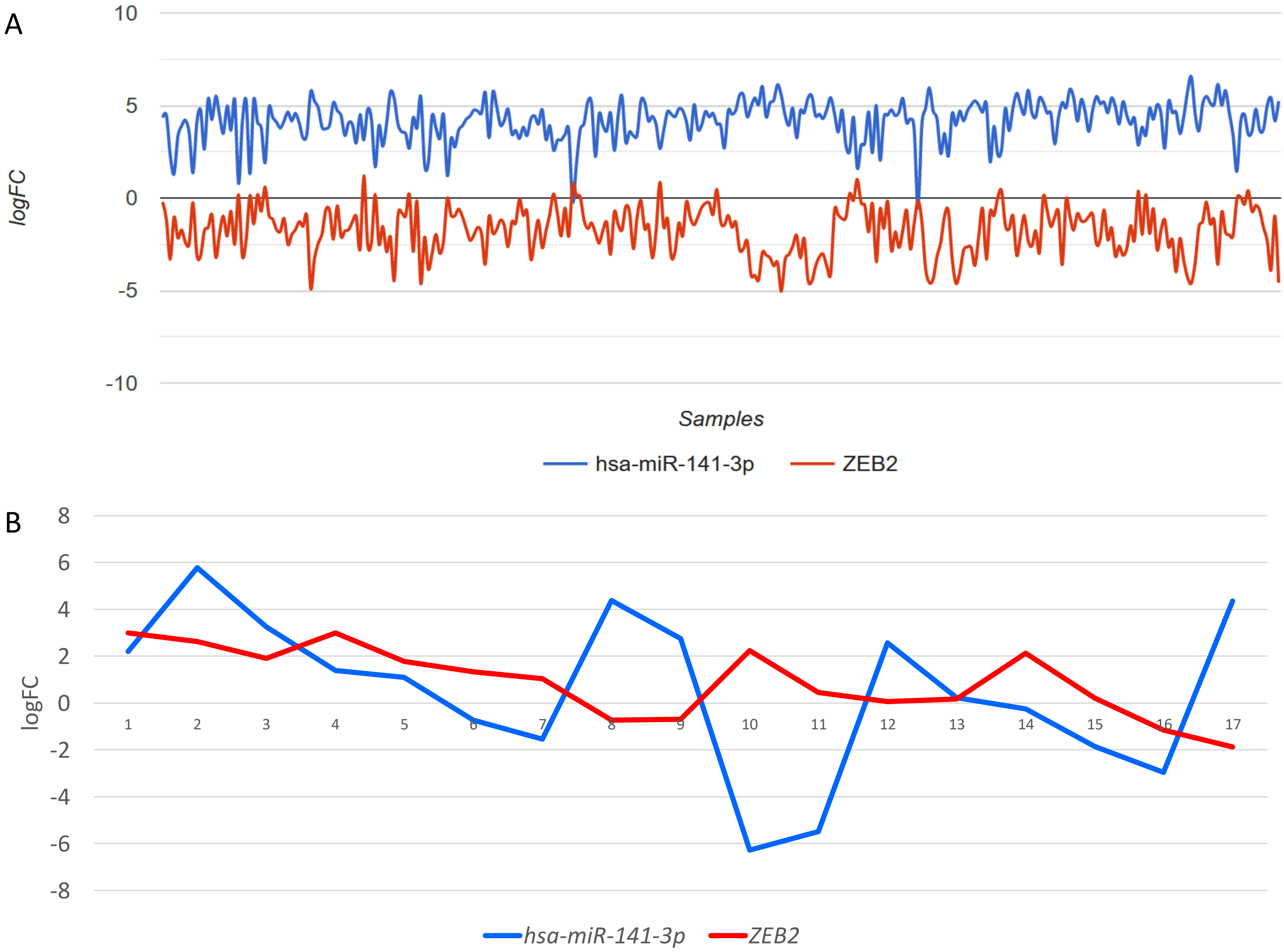
A
10
5
0
-5
-10
logFC
Samples
hsa-miR-141-3p
ZEB2
B
8
6
4
2
0
-2
-4
-6
-8
logFC
1
2
3
4
5
6
7
8
9
10
11
12
13
14
15
16
17
hsa-miR-141-3p
ZEB2

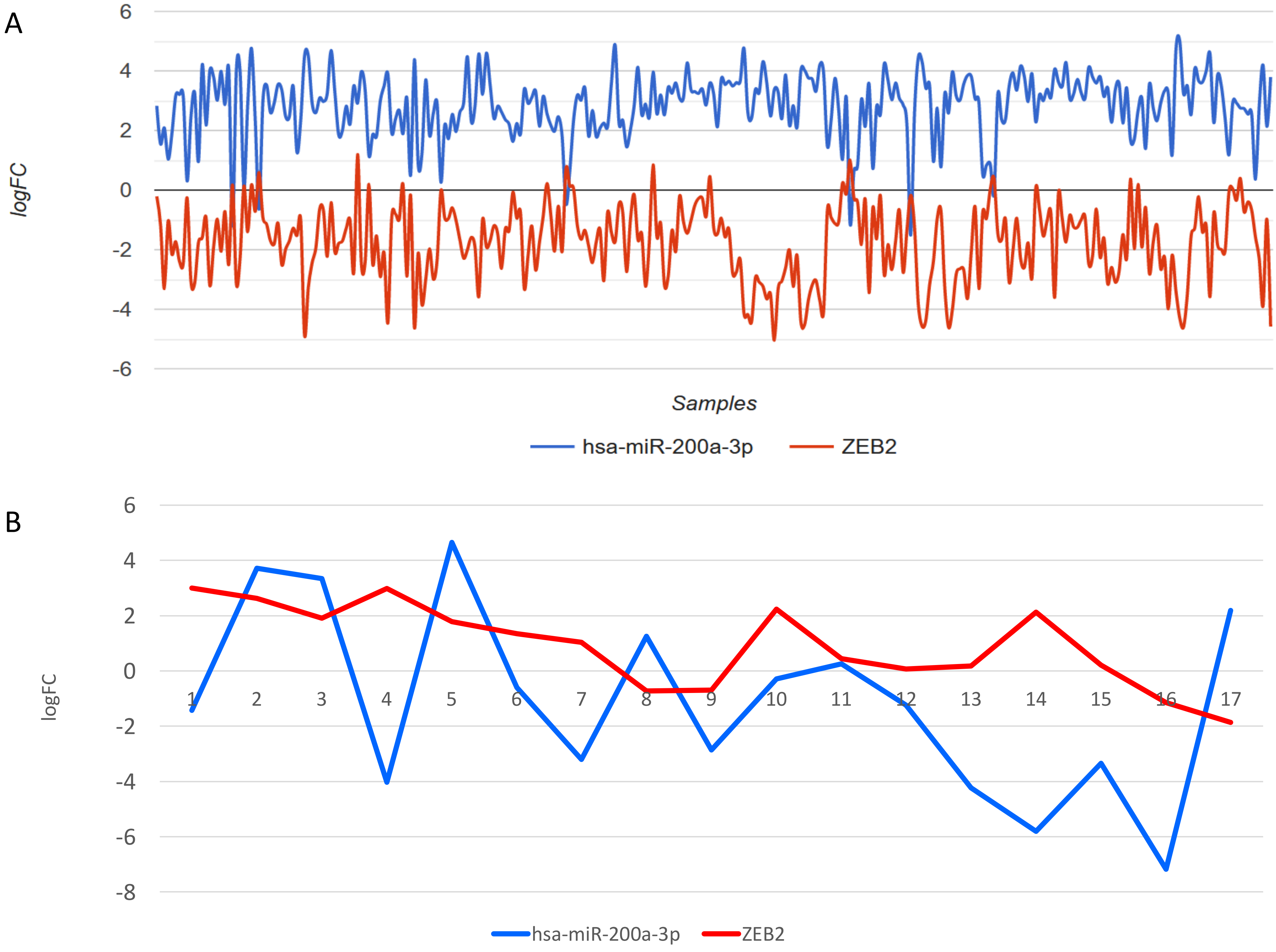
A
6
4
2
0
-2
-4
-6
logFC
Samples
hsa-miR-200a-3p
ZEB2
B
6
4
2
0
-2
-4
-6
-8
logFC
1
2
3
4
5
6
7
8
9
10
11
12
13
14
15
16
17
hsa-miR-200a-3p
ZEB2

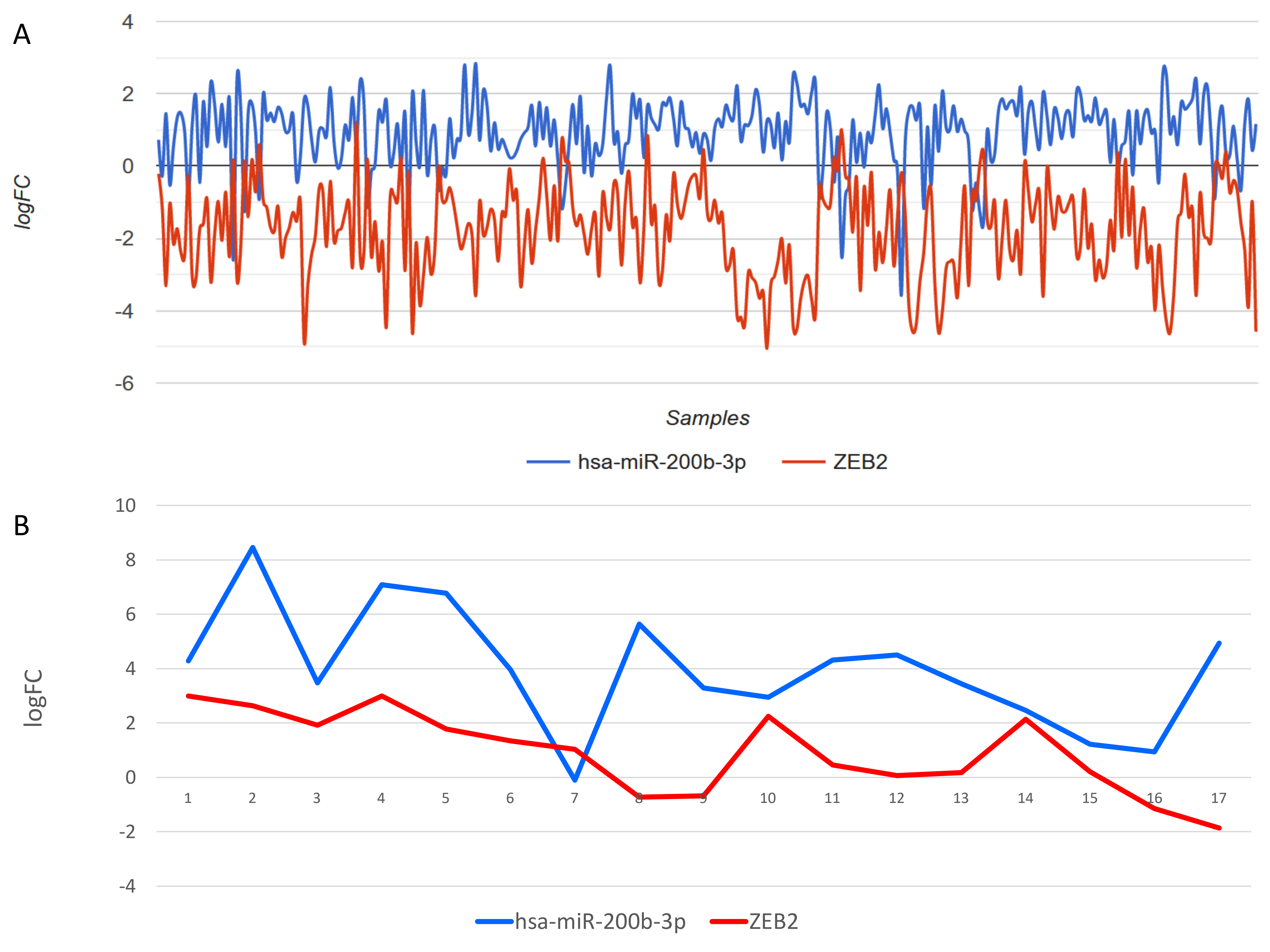

A
4
2
0
-2
-4
-6
logFC
Samples
hsa-miR-200b-3p
ZEB2
B
10
8
6
4
2
0
-2
-4
logFC
1 2 3 4 5 6 7 8 9 10 11 12 13 14 15 16 17
hsa-miR-200b-3p
ZEB2

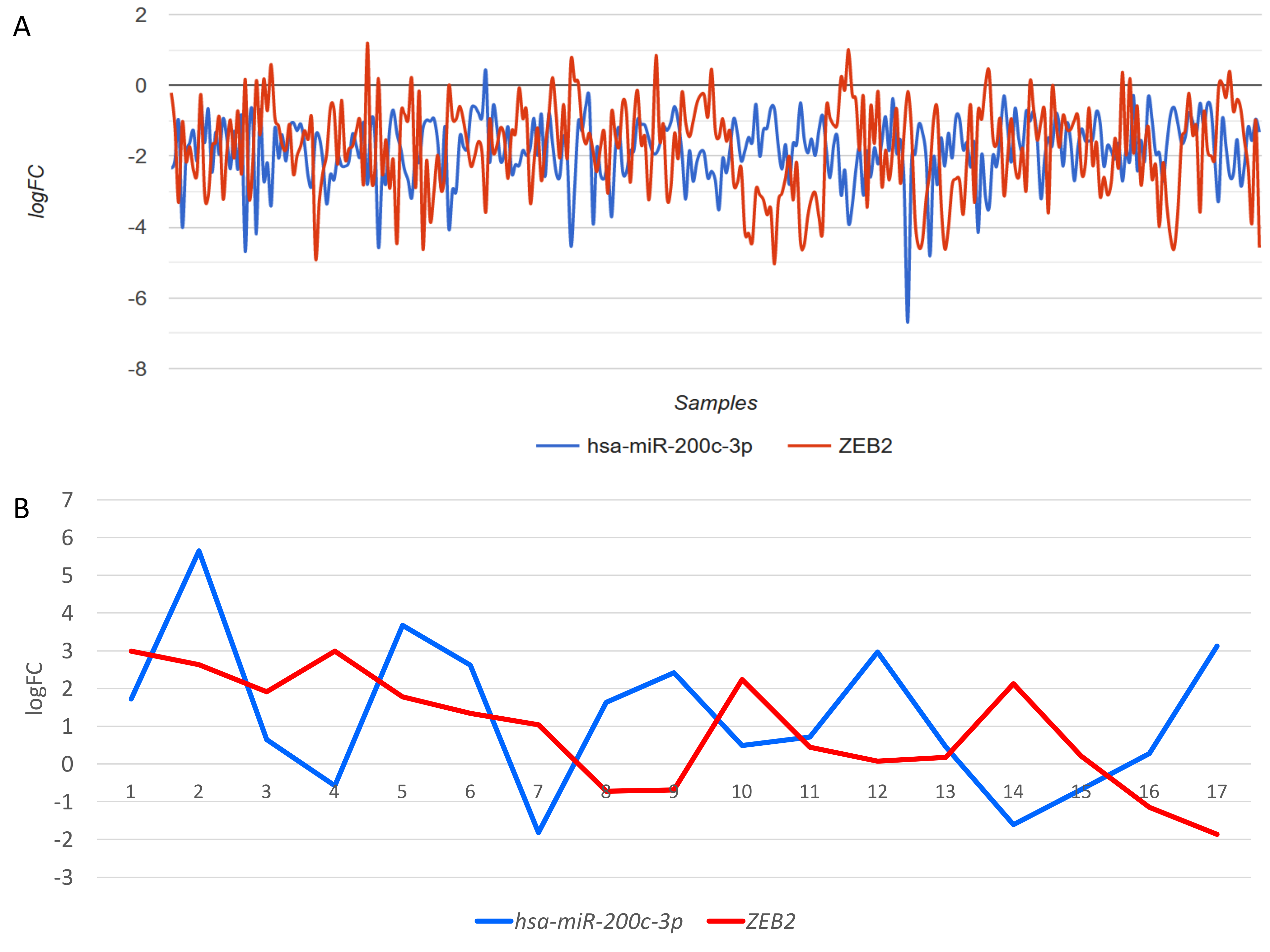

A
2
0
-2
-4
-6
-8
logFC
Samples
hsa-miR-200c-3p
ZEB2
B
7
6
5
4
3
2
1
0
-1
-2
-3
logFC
1
2
3
4
5
6
7
8
9
10
11
12
13
14
15
16
17
hsa-miR-200c-3p
ZEB2

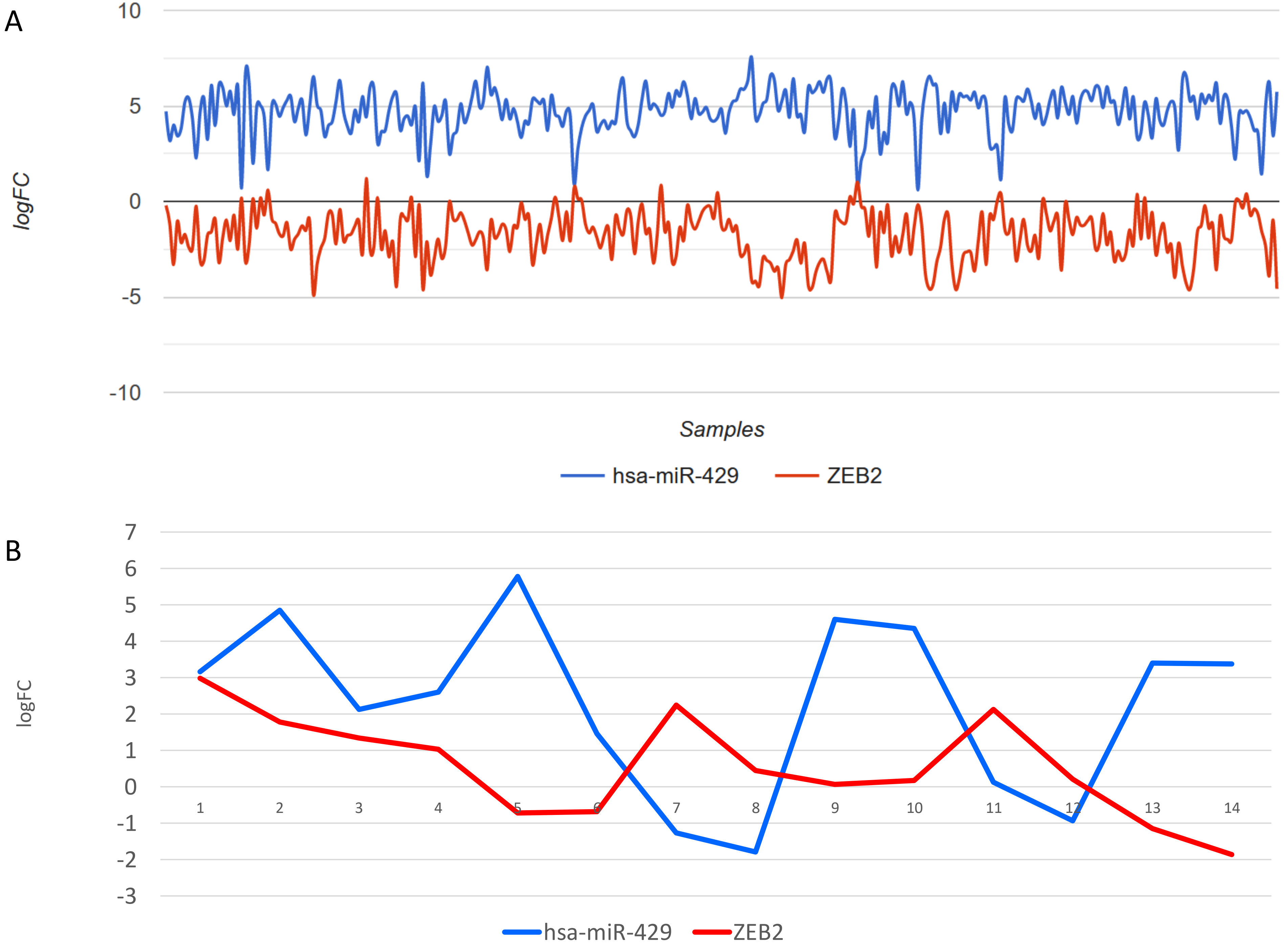
A
10
5
0
-5
-10
logFC
Samples
hsa-miR-429
ZEB2
B
7
6
5
4
3
2
1
0
-1
-2
-3
logFC
1
2
3
4
5
6
7
8
9
10
11
12
13
14
hsa-miR-429
ZEB2

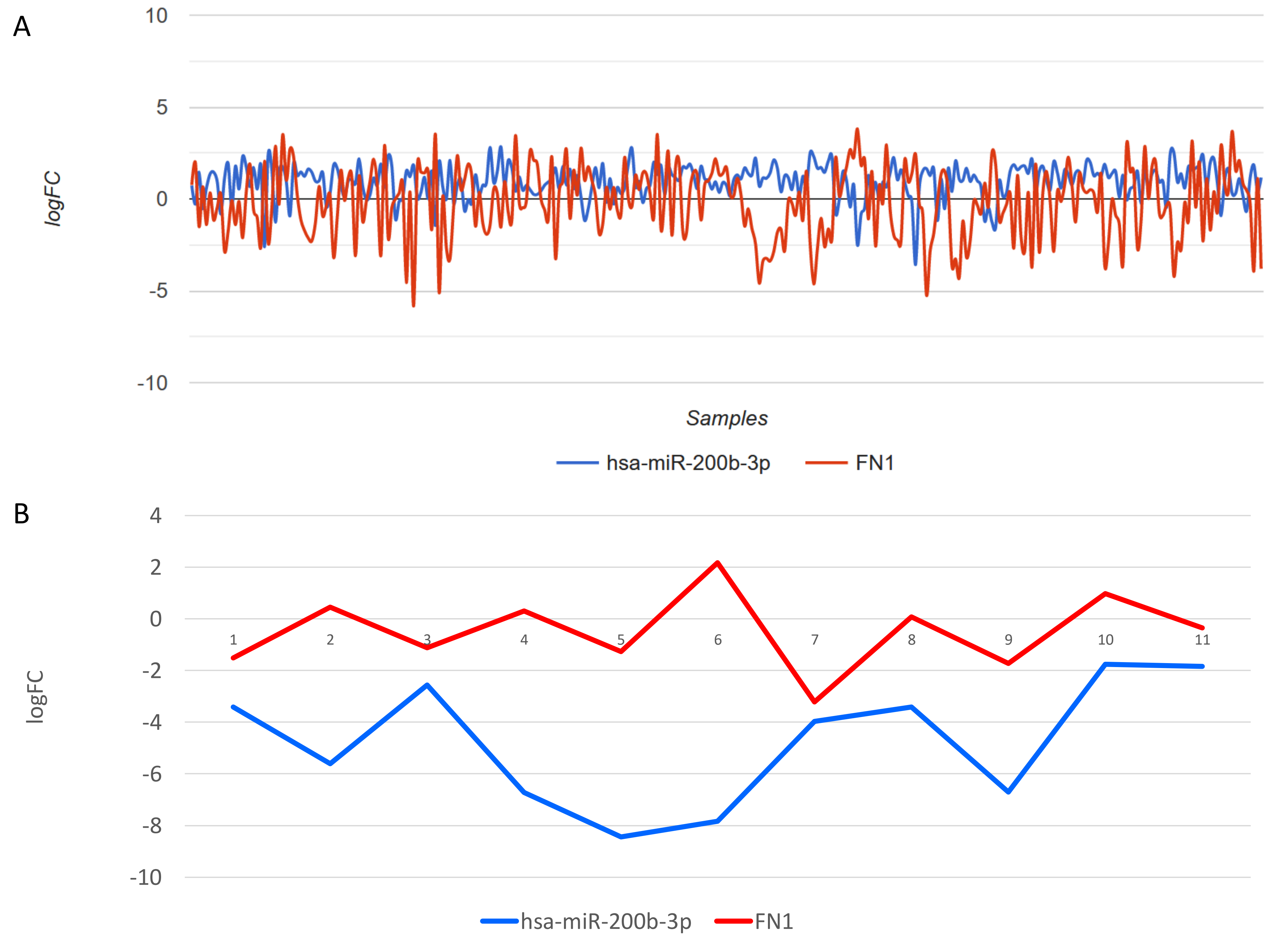
A
10
5
0
-5
-10
logFC
Samples
hsa-miR-200b-3p
FN1
B
4
2
0
-2
-4
-6
-8
-10
logFC
1
2
3
4
5
6
7
8
9
10
11
hsa-miR-200b-3p
FN1

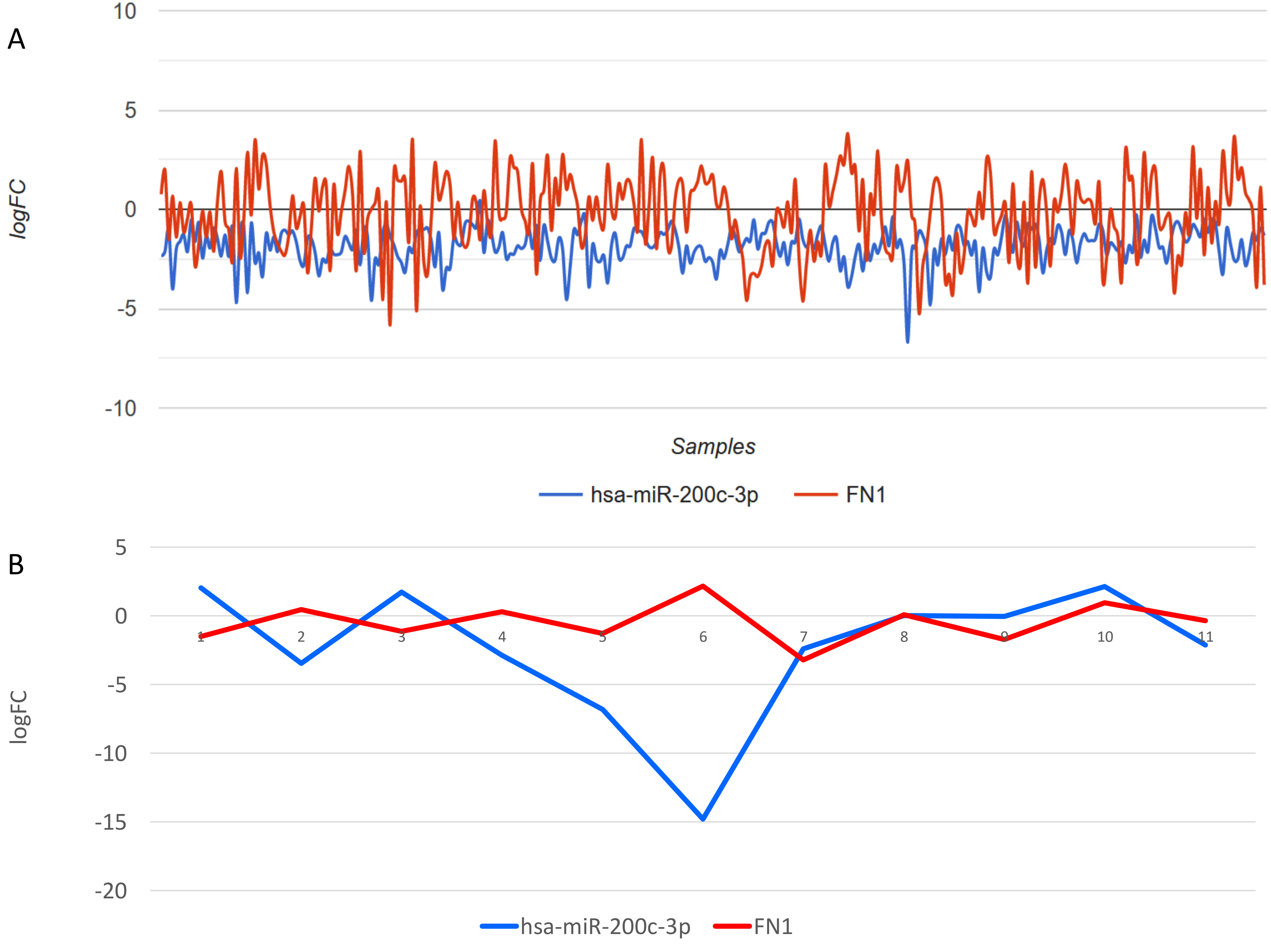
A
10
5
0
-5
-10
logFC
Samples
hsa-miR-200c-3p
FN1
B
5
0
-5
-10
-15
-20
logFC
1
2
3
4
5
6
7
8
9
10
11
hsa-miR-200c-3p
FN1

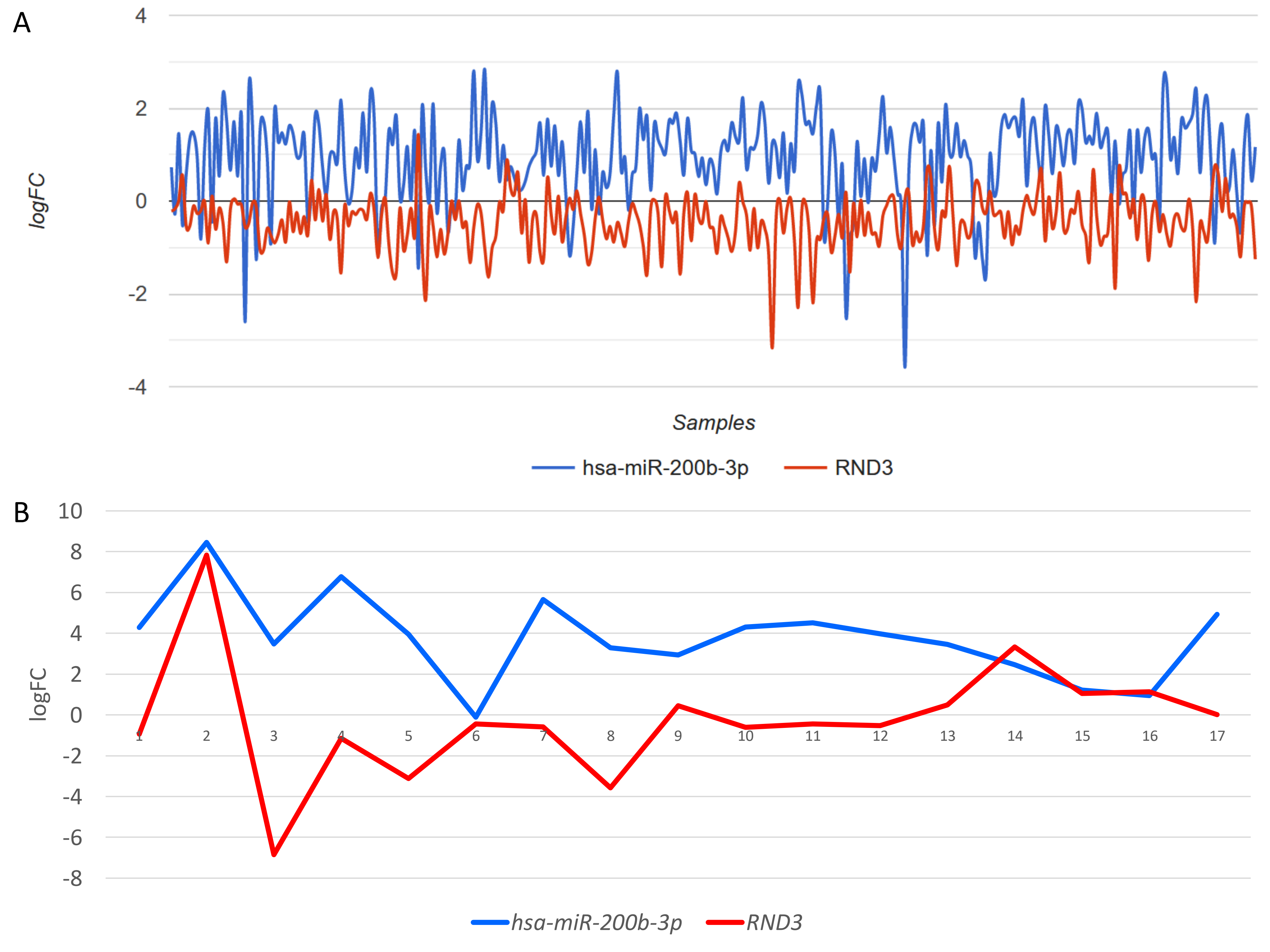
A
4
2
0
-2
-4
logFC
Samples
hsa-miR-200b-3p
RND3
B
10
8
6
4
2
0
-2
-4
-6
-8
logFC
1
2
3
4
5
6
7
8
9
10
11
12
13
14
15
16
17
hsa-miR-200b-3p
RND3

Supplementary material

Supplementary Table 1. Table of expression values of miRNA-target gene pairs and their correlations.

Supplementary Table 2. Table of reads per million kilobases of miRNA isoforms for all samples used in the analysis.

Supplementary Figure 1. Distributions of miRNA-mRNA pairs: (A) from our bioinformatics analysis and (B) from our experimental results. Figures represent miRNA-target gene pairs: *TGFB2*/*has-miR-141-3p*, *TGFB2*/*has-miR-200a-3p*, *ZEB2*/*has-miR-141-3p*, *ZEB2*/*has-miR-200a-3p*, *ZEB2*/*has-miR-200b-3p*, *ZEB2*/*has-miR-200c-3p*, *ZEB2*/*has-miR-429*, *FN1*/*has-miR-200b-3p*, *FN1*/*has-miR-200c-3p* and *RND3*/*has-miR-200b-3p*.
